# Survival and clinical prognostic factors in metastatic non‐clear cell renal cell carcinoma treated with targeted therapy: A multi‐institutional, retrospective study using the Korean metastatic renal cell carcinoma registry

**DOI:** 10.1002/cam4.2222

**Published:** 2019-05-09

**Authors:** Jung Kwon Kim, Sung Han Kim, Mi Kyung Song, Jungnam Joo, Seong Il Seo, Cheol Kwak, Chang Wook Jeong, Cheryn Song, Eu Chang Hwang, Ill Young Seo, Hakmin Lee, Sung‐Hoo Hong, Jae Young Park, Jinsoo Chung

**Affiliations:** ^1^ Department of Urology Seoul National University Bundang Hospital Seongnam Republic of Korea; ^2^ Department of Urology, Center for Prostate Cancer National Cancer Center Goyang Republic of Korea; ^3^ Biometric Research Branch, Center for Prostate Cancer National Cancer Center Goyang‐si Republic of Korea; ^4^ Department of Urology Sungkyunkwan University College of Medicine Seoul Republic of Korea; ^5^ Department of Urology Seoul National University College of Medicine Seoul Republic of Korea; ^6^ Department of Urology University of Ulsan College of Medicine Seoul Republic of Korea; ^7^ Department of Urology, Medical School Chonnam National University Hwasun‐gun Republic of Korea; ^8^ Department of Urology Wonkwang University School of Medicine and Hospital Iksan Republic of Korea; ^9^ Department of Urology Seoul St. Mary's Hospital, The Catholic University Seoul Republic of Korea; ^10^ Department of Urology Korea University Ansan Hospital, Korea University College of Medicine Ansan Republic of Korea

**Keywords:** Korean, metastatic renal cell carcinoma, non‐clear cell, prognosis, survival

## Abstract

**Objectives:**

The optimal treatment strategy for metastatic non‐clear cell renal cell carcinoma (mNCCRCC) is still elusive and mainly extrapolated from evidence available for metastatic clear cell renal cell carcinoma. The aim of the study was therefore to investigate the survival outcomes and prognostic factors affecting survival in patients with mNCCRCC treated with targeted therapy.

**Materials and methods:**

We analyzed a total of 156 patients (8.1%) with mNCCRCC among the total cohort of 1922 patients in the Korean metastatic RCC registry. We used Kaplan‐Meier curve analysis to calculate the survival estimates for first‐line progression‐free survival (PFS), total PFS, and cancer‐specific survival (CSS). We also used the log‐rank test to compare the different groups and multivariate Cox‐proportional hazard regression analyses to evaluate the prognostic factors for survival.

**Results:**

The mNCCRCC group had significantly inferior survival outcomes in terms of first‐line PFS, total PFS, and CSS (all *P* < 0.05). We found survival benefits in patients treated with first‐line vascular endothelial growth factor‐tyrosine kinase inhibitors (VEGF‐TKIs, first‐line PFS, and total PFS, all *P* < 0.05), cytoreductive nephrectomy (CSS, *P* < 0.0001), metastasectomy (CSS, *P* = 0.0017), and patients with metachronous metastasis (first‐line PFS, total PFS, and CSS, all *P* < 0.05). Liver metastasis was the only significant prognostic factor for both first‐line PFS and CSS (all *P* < 0.05).

**Conclusions:**

In the current targeted therapy era, survival of mNCCRCC is still inferior in comparison with that of mCCRCC patients. We found survival benefits in patients treated with first‐line VEGF‐TKIs/CN/metastasectomy, and metachronous metastasis patients.

## 1 INTRODUCTION

In field of urologic cancer, renal cell carcinoma (RCC) is a common malignancy with the annual diagnosis of over 65 000 cases in the United States.^1^ Among these RCCs, metastasis was observed in 20%‐30% of cases during follow‐up, even in localized RCCs underwent curative treatment.^2^ According to the 2004 World Health Organization (WHO) classification system,^3^ the most common type of RCC is clear cell RCC (CCRCC, 70%‐85%). The remaining subtypes comprise papillary, chromophobe, collecting duct, unclassified, and Xp11.2 transposition; they are often classified as non‐clear cell RCC (NCCRCC).

Metastatic NCCRCC (mNCCRCC) includes heterogeneous subgroups that profoundly differ in terms of morphology, genetic profile, clinical characteristic, and prognosis. The optimal treatment strategy for mNCCRCC is still elusive and mainly extrapolated from evidence available for mCCRCC. Thus, the optimal treatment strategy is represented by novel agents that target the members of vascular endothelial growth factor (VEGF) and the mammalian target of rapamycin (mTOR) pathways.^4^ Due to the rarity of NCCRCC tumors, only a few prospective randomized trials have been reported so far.^5^, ^6^ Consequently, the efficacy of targeted therapy (TT) remains largely unknown in the field of mNCCRCC.

The impact of ethnic differences between Asian and non‐Asian populations in drug absorption and metabolism have been well established.^7^, ^8^ However, these pharmacoethnic differences in response to TT are still not well recognized.^9^, ^10^ Guo et al^9^ reported a distinct pattern and severity of adverse events between Asian and non‐Asian patients in the subgroup analysis of the COMPARZ trial. The more prevalent adverse events were hematologic toxicity and cytopenia in Asian patients, whereas gastrointestinal toxicity was more prevalent in non‐Asian patients.

Regarding an Asian population, the incidence of RCC is lower compared to worldwide data^11^; however, the annual percentage change in RCC has been increasing gradually in Korea.^12^ In addition, there were 11.3% of patients with metastatic disease at the time of diagnosis in 2014 among these cases.

However, the majority of studies had been conducted considering mCCRCC, the predominant histologic subtype in the study cohorts. The aim of the study was therefore to investigate the survival outcomes and clinical prognostic factors affecting survival in Korean patients with mNCCRCC treated with TT.

## 2 METHODS

### 2.1 Study cohort

The detailed description of the Korean metastatic RCC registry has been reported in our previous study.^13^ We analyzed the data of 1922 patients who had received TT (VEGF‐tyrosine kinase inhibitors [VEGF‐TKIs], mTOR inhibitor [mTORi]) or cytokines as first‐line treatments between 2001 and 2016. All institutions included in the study received institutional review board approval before inputting data into the registry. For consistent data collection, unified data templates were used at each institution. We retrospectively reviewed medical records and/or death certificate data to analyze survival data. As this study was carried out retrospectively, written informed consent from patients was waived. Personal identifiers were completely removed and the data were analyzed anonymously.

### 2.2 Acquisition and definition of data

The following clinicopathologic variables were collected: age at diagnosis, sex, Karnofsky performance status, Eastern Cooperative Oncology Group performance status, histologic subtypes, first‐line treatment agents, the International Metastatic Renal Cell Carcinoma Database Consortium (IMDC),^14^, ^15^ Memorial Sloan Kettering Cancer Center (MSKCC)^16^ risk group, metastasis details (site, synchronous, or metachronous), cytoreductive nephrectomy (CN), and metastasectomy.

Synchronous lesions were considered as metastases diagnosed at the time of diagnosis or within 3 months after primary nephrectomy; metachronous lesions were defined as recurring or progressed localized RCC that has metastasized after curative surgical treatment. Progression was defined according to radiographic criteria using the Response Evaluation Criteria in Solid Tumors (RECIST, version 1.1).^17^ We continued treatment until disease progression was detected or intolerable adverse events were reported. First‐line progression‐free survival (PFS) was defined as the period between the date of the first treatment and the progressive disease, and cancer‐specific survival (CSS) as between the date of the first treatment and RCC‐related death or the last follow‐up visit. Total PFS was defined as the sum of the first line PFS and subsequent treatment PFS.

### 2.3 Statistical analyses

We used the Kaplan‐Meier curve analysis to calculate survival estimates for first‐line PFS, total PFS, and CSS. Also, we used the log‐rank test to compare the different groups: CCRCC versus NCCRCC (including all histologic subtypes), VEGF‐TKIs versus mTORi as the first‐line treatment, synchronous versus metachronous metastasis as the metastatic type, use of CN, and metastasectomy. We performed univariate and multivariate Cox‐proportional hazard regression analyses to evaluate the significant variables associated with the first‐line PFS, total PFS, and CSS. We considered a two‐sided p value of less than 0.05 to be statistically significant, and we performed all statistical analyses using SAS statistical software (version 9.4; SAS Institute Inc, Cary, NC) and R‐project software (version 3.3.3).

## 3 RESULTS

The baseline characteristics are summarized in our previous study^13^ and supplemental Table S1 of the current study. A total of 156 (8.1%) patients with mNCCRCC were analyzed; these comprised 93 (59.6%) papillary, 20 (12.8%) chromophobe, 18 (11.5%) collecting duct, 16 (10.3%) unclassified, and nine (5.8%) Xp11.2 translocation RCC. Regarding the first‐line therapy, majority of patients (91.0%) were treated with TT, while only 14 patients (9.0%) were treated with cytokine immunotherapy. Lymph nodes (53.3%) were reported as the most common site of metastasis, followed by the lung (46.1%), bone (42.9%), liver (26.6%), and brain (3.3%).

**Figure 1:**
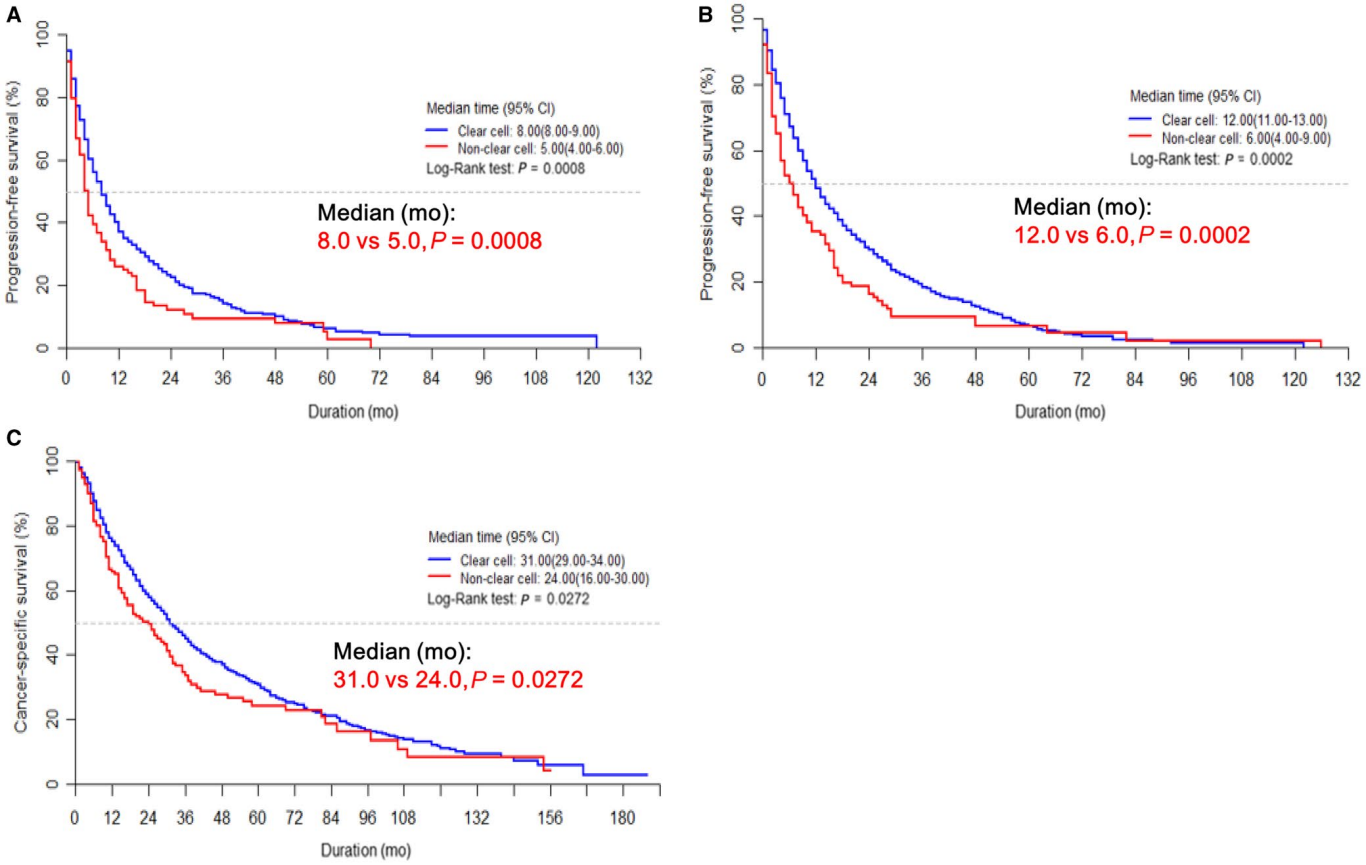
Kaplan‐Meier survival curves for (A) first‐line progression‐free survival (PFS), (B) total PFS, and (C) cancer‐specific survival (CSS) for patients with clear cell and non‐clear cell metastatic renal cell carcinoma

The survival outcomes for the mNCCRCC group were significantly inferior to those for the mCCRCC group in terms of first‐line PFS (median: 5.0 vs 8.0 months, *P* = 0.0008), total PFS (median: 6.0 vs 12.0 months, *P* = 0.0002), and CSS (median: 24.0 vs 31.0 months, *P* = 0.0272; Figure 1).

### 3.1 Comparison of survival outcomes according to the histologic subtypes

**Figure 2:**
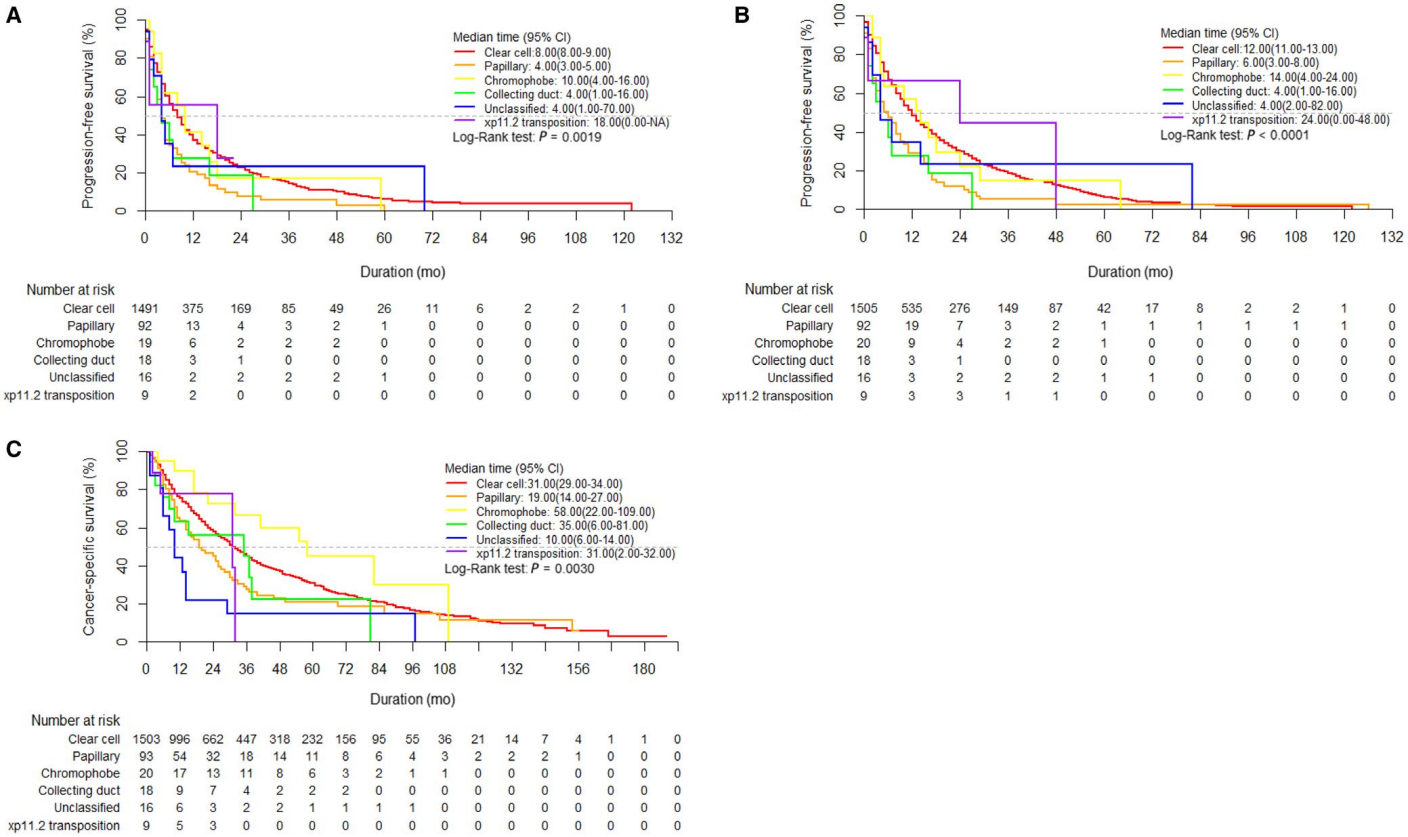
Kaplan‐Meier survival curves for (A) first‐line progression‐free survival (PFS), (B) total PFS, and (C) cancer‐specific survival (CSS) according to the histologic types

Kaplan‐Meier survival analysis showed significantly favorable survival outcomes in terms of first‐line PFS (median: 10.0 vs 18.0 vs 8.0 months), total PFS (median: 14.0 vs 24.0 vs 12.0 months), and CSS (median: 58.0 vs 31.0 vs 31.0 months, all respectively) in the chromophobe and Xp11.2 transposition groups compared to the clear cell group (Figure 2 and Supplemental Table S2). Conversely, survival outcomes of the papillary, collecting duct, and unclassified groups were significantly poorer than those of the clear cell group (first‐line PFS, median: 4.0 vs 4.0 vs 4.0 vs 8.0 months; total PFS, median: 6.0 vs 4.0 vs 4.0 vs 12.0 months; CSS, median: 19.0 vs 35.0 vs 10.0 vs 31.0 months, all respectively).

### 3.2 Comparison of survival outcomes according to the first‐line treatment

**Figure 3:**
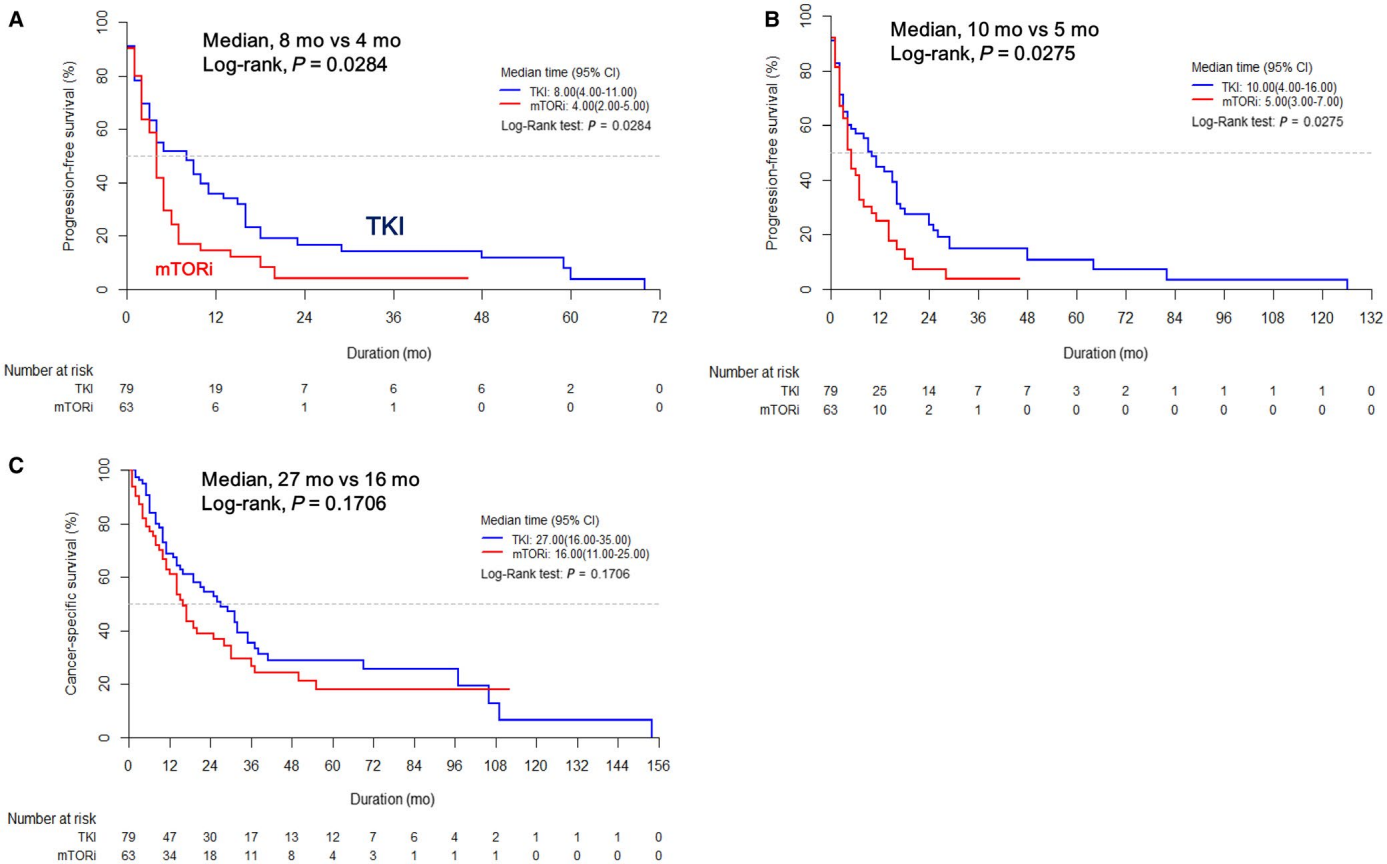
Kaplan‐Meier survival curves for (A) first‐line progression‐free survival (PFS), (B) total PFS, and (C) cancer‐specific survival (CSS) according to the first‐line treatment

Kaplan‐Meier survival analysis showed that the VEGF‐TKIs group (n = 79, 50.6%) had significantly better survival outcomes than the mTORi group (n = 63, 40.4%) in terms of first‐line PFS (median: 8.0 vs 4.0 months, *P* = 0.0284) and total PFS (median: 10.0 vs 5.0 months, *P* = 0.0275). However, regarding CSS, no statistically significant difference was shown between the two groups (median: 27.0 vs 16.0 month, *P* = 0.1706; Figure 3).

### 3.3 Comparison of survival outcomes according to the type of metastasis

**Figure 4:**
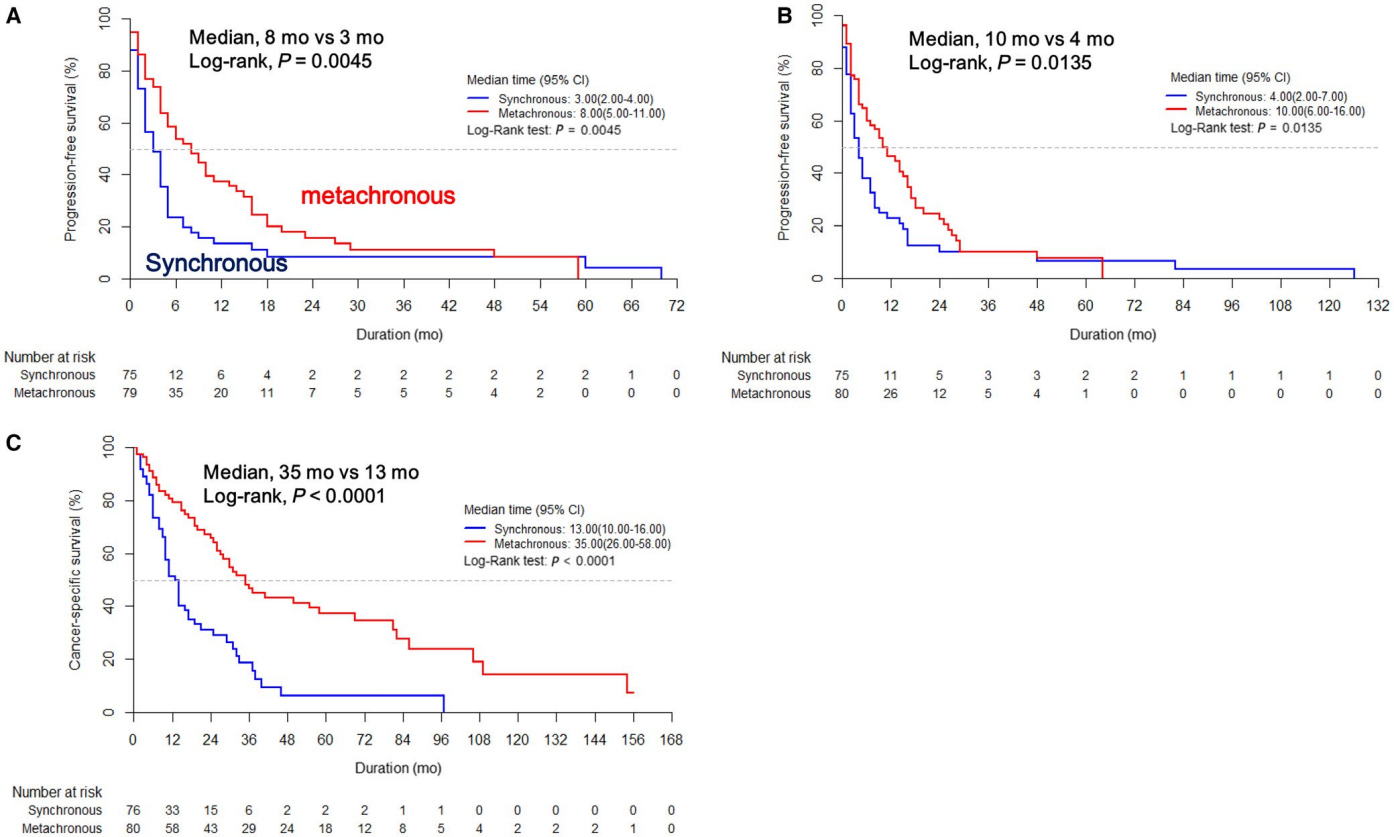
Kaplan‐Meier survival curves for (A) first‐line progression‐free survival (PFS), (B) total PFS, and (C) cancer‐specific survival (CSS) according to the metastatic types

Kaplan‐Meier survival analysis showed that the survival outcomes the metachronous group were significantly better than those of the synchronous group in terms of first‐line PFS (median: 8.0 vs 3.0 months, *P* = 0.0045), total PFS (median: 10.0 vs 4.0 months, *P* = 0.0135), and CSS (median: 35.0 vs 13.0 months, *P* < 0.0001; Figure 4).

### 3.4 Comparison of survival outcomes according to the use of CN and metastasectomy

Kaplan‐Meier survival analysis showed that the CSS of the CN group was significantly better than that of the non‐CN group (median: 30.0 vs 6.0 months, *P* < 0.0001). However, no statistically significant differences were found between the two groups in terms of first‐line PFS (median: 5.0 vs 2.0 months, *P* = 0.0915) and total PFS (median: 7.0 vs 2.0 months, *P* = 0.3630; Supplemental Figure S1). In addition, while the metastasectomy group also showed significantly better survival outcomes than the non‐metastasectomy group in terms of CSS (median: 81.0 vs 17.0 months, *P* = 0.0017), they were not significantly better in first‐line PFS (median: 7.0 vs 4.0 months, *P* = 0.1325) and total PFS (median: 7.0 vs 6.0 months, *P* = 0.1652; Supplemental Figure S2).

### 3.5 Prognostic factors for survival

Multivariable Cox regression analysis revealed that metachronous metastasis (hazard ratio [HR]: 0.593; 95% confidence interval [CI]: 0.357‐0.986, *P* = 0.0442) and liver metastasis (HR: 1.856; 95% CI: 1.228‐2.805, *P* = 0.0033) were significant prognostic factors for first‐line PFS (Table 1). The significant prognostic factors for CSS were both the MSKCC (HR: 2.689; 95% CI: 1.116‐6.387) and IMDC (HR: 2.919; 95% CI: 1.270‐6.637) poor risk groups, lung (HR: 2.135; 95% CI: 1.183‐3.818), liver (HR: 3.868; 95% CI: 1.713‐8.205), and bone (HR: 1.630; 95% CI: 1.057‐2.515) metastasis (*P* < 0.05; Table 2).

**Table 1:** Cox regression analysis for first‐line progression‐free survival

	Univariate		Multivariate	
	HR (95% CI)	*P*	HR (95% CI)	*P*
Histology				
Papillary	1 (Ref)			
Chromophobe	0.612 (0.337‐1.110)	0.1059		
Collecting duct	0.975 (0.515‐1.846)	0.9381		
Unclassified	0.637 (0.303‐1.340)	0.2348		
Xp11.2 translocation	0.659 (0.265‐1.639)	0.3693		
Type of first‐line therapy				
VEGF‐TKIs	1 (Ref)			
mTORi	0.584 (0.354‐0.820)	0.0324	0.629 (0.410‐1.184)	0.1954
Metastasis type				
Synchronous	1 (Ref)		1 (Ref)	
Metachronous	0.591 (0.400‐0.871)	0.0080	0.593 (0.357‐0.986)	0.0442
MSKCC risk group				
Favorable	1 (Ref)		1 (Ref)	
Intermediate	1.250 (0.799‐1.955)	0.3280	0.989 (0.576‐1.699)	0.9689
Poor	2.885 (1.426‐5.836)	0.0032	1.948 (0.827‐4.588)	0.1270
IMDC risk group				
Favorable	1 (Ref)		1 (Ref)	
Intermediate	1.276 (0.812‐2.004)	0.2902	1.009 (0.587‐1.735)	0.9734
Poor	2.879 (1.520‐5.454)	0.0012	2.118 (0.980‐4.578)	0.0563
Lung metastasis, yes	0.823 (0.561‐1.208)	0.3202		
Liver metastasis, yes	1.639 (1.094‐2.457)	0.0167	1.856 (1.228‐2.805)	0.0033
L/N metastasis, yes	1.095 (0.745‐1.609)	0.6453		
Bone metastasis, yes	0.888 (0.600‐1.313)	0.5507		
Brain metastasis, yes	0.684 (0.245‐1.911)	0.4687		
Cytoreductive nepherectomy, yes	0.566 (0.225‐1.423)	0.2263		
Metastasectomy, yes	0.707 (0.437‐1.142)	0.1562		

**Table 2:** Cox regression analysis for cancer‐specific survival

	Univariate		Multivariate	
	HR (95% CI)	*P*	HR (95% CI)	*P*
Histology				
Papillary	1 (Ref)		1 (Ref)	
Chromophobe	0.484 (0.254‐0.920)	0.0268	0.629 (0.310‐1.174)	0.1754
Collecting duct	0.993 (0.523‐1.887)	0.9829	0.725 (0.326‐1.483)	0.4060
Unclassified	1.721 (0.945‐3.134)	0.0757	1.587 (0.717‐3.298)	0.2370
Xp11.2 translocation	0.811 (0.294‐2.238)	0.6865	0.581 (0.171‐1.574)	0.3322
Type of first‐line therapy				
VEGF‐TKIs	1 (Ref)			
mTORi	0.729 (0.410‐1.221)	0.1853		
Metastasis type				
Synchronous	1 (Ref)		1 (Ref)	
Metachronous	0.383 (0.255‐0.577)	<0.0001	0.583 (0.325‐1.047)	0.0708
MSKCC risk group				
Favorable	1 (Ref)		1 (Ref)	
Intermediate	1.762 (1.086‐2.859)	0.0218	1.199 (0.675‐2.142)	0.5425
Poor	4.911 (2.536‐9.510)	<0.0001	2.689 (1.116‐6.387)	0.0277
IMDC risk group				
Favorable	1 (Ref)		1 (Ref)	
Intermediate	1.779 (1.088‐2.909)	0.0217	1.184 (0.662‐2.143)	0.5757
Poor	4.894 (2.578‐9.289)	<0.0001	2.919 (1.270‐6.637)	0.0118
Lung metastasis, yes	2.279 (1.433‐3.623)	0.0005	2.135 (1.183‐3.818)	0.0116
Liver metastasis, yes	3.492 (1.724‐7.073)	0.0005	3.868 (1.713‐8.205)	0.0007
L/N metastasis, yes	1.944 (1.256‐3.009)	0.0028	1.474 (0.752‐2.950)	0.2668
Bone metastasis, yes	1.585 (1.070‐2.347)	0.0215	1.630 (1.057‐2.515)	0.0271
Brain metastasis,	0.576 (0.142‐2.340)	0.4408		
Cytoreductive nepherectomy, yes	0.788 (0.607‐0.898)	<0.0001	0.942 (0.743‐1.180)	0.1190
Metastasectomy, yes	0.430 (0.249‐0.744)	0.0026	0.570 (0.318‐1.021)	0.0586

## 4 DISCUSSION

In an Asian population, this is the largest nationwide study to investigate mNCCRCC treated with TT until now. Importantly, we found that the survival outcomes of the mNCCRCC patients were inferior to those of the mCCRCC patients in the current TT era (Figure 1). Several previous studies also reported similar results regarding this issue.^18^, ^19^ Upton et al^18^ reported that mNCCRCC showed a worse response to conventional immunotherapies than mCCRCC (overall response rate 21% vs 6%, respectively). In a recent large retrospective analysis of IMDC, Kroeger et al^19^ found that mNCCRCC patients had significantly poorer overall survival (OS) than did patients with mCCRCC treated with TT agents (12.8 vs 22.3 months; *P* < 0.0001); chromophobe RCC had the best OS, and those with papillary and unclassified RCC had the worst OS. In concordance with their study, we also found significantly favorable survival outcomes in the chromophobe and Xp11.2 transposition groups compared to the clear cell group; conversely, the survival outcomes of the papillary, collecting duct, and unclassified group were significantly poorer than those of the clear cell group. With this perspective, we tentatively suggest that we need to focus more on each subtype to evaluate the individual survival outcomes.

In the field of mNCCRCC, the results of two prospective phase II randomized trials (ESPN and ASPEN) comparing the activity of the VEGF‐TKIs (sunitinib) with the mTORi (everolimus) were reported^5^, ^6^; both studies demonstrated better PFS in the first‐line sunitinib group than in the everolimus group (6.1 vs 4.1 months [ESPN] and 8.3 vs 5.6 months [ASPEN], respectively). However, regarding OS, the VEGF‐TKIs were not superior to mTORi. Several hypotheses (including impact of subsequent treatment,^20^ type of the VEGF‐TKi and its spectrum of molecular targets^21^) have been proposed to explain the non‐superiority in OS, although none of these was conclusive. Regarding the other mTORi (temsirolimus), in the previous phase III ARCC trial, Hudes et al^22^ reported that first‐line temsirolimus offer an OS advantage compared with interferon alfa (HR: 0.73; 95% CI: 0.58‐0.92; *P* = 0.008) in patients with metastatic RCC and a poor prognosis. In their study, they included a total of 40 mNCCRCC patients; this is still the largest study evaluating the efficacy of temsirolomus as first‐line treatment in mNCCRCC.

Importantly, in the current study, the majority of patients (n = 47, 74.6%) in the mTORi group received temsirolimus as first‐line treatment (Supplemental Table S1). This reflects the largest study to date that compares the efficacy of temsirolimus with VEGF‐TKIs in mNCCRCC patients as a first‐line treatment. In concordance with previous studies,^5^, ^6^ we also found that the VEGF‐TKI group had significantly better first‐line (8.0 vs 4.0 months) and total (10.0 vs 5.0 months) PFS than the mTORi group, but not CSS (Figure 3). Consequently, we tentatively concluded that the first‐line VEGF‐TKIs is, at present, the most recommended treatment option in patients with mNCCRCC, supporting the standard treatment paradigm broadly used for mCCRCC patients.

Generally, metachronous metastasis (MM) is considered to have a better prognosis than synchronous metastasis (SM) in the field of metastatic RCC.^23^, ^24^ It can be explained by the presence or absence of primary tumors following nephrectomy, or the distinct phenotypes with different ontogenetic activities between the two groups. Kim et al^24^ reported that MM is associated with more favorable survival outcomes than SM in patients with metastatic RCC treated with VEGF‐TKIs (OS, 20.1 vs 9.6 months, *P* = 0.010). In the current study, we also found that that MM had significantly better survival outcomes than SM in Kaplan‐Meier survival analysis in our mNCCRCC cohort (Figure 4). However, in multivariate analysis, metastatic type was only a statistically significant prognostic factor in the first‐line PFS, not in the CSS (Tables 1 and 2). This might be due to the small number of cases and uncontrolled heterogeneity of various degrees of tumor burden. Thus, further larger studies are warranted.

Regarding the surgical treatment in the field of metastatic RCC, the use of CN in patients treated with conventional immunotherapy is evident.^25^ Also, in a previous prospective study, Daliani et al^26^ evaluated the value of metastasectomy in patients receiving cytokine, tumor vaccine, and/or chemotherapy; they found that 29 (76%) of the 38 patients enrolled showed no evidence of disease postoperatively, and the median PFS and OS were 1.8 and 4.7 years, respectively. However, the role of CN and metastasectomy in the area of TT still needs to be defined. Recently, the randomized phase III CARMENA trial showed that sunitinib alone was not inferior to CN followed by sunitinib in patients with mCCRCC who were classified as having intermediate‐risk or poor‐risk disease (HR: 0.89; 95% CI: 0.71 to 1.10; upper boundary of the 95% CI for noninferiority, ≤1.20).^27^ The median OS for patients who received the sunitinib alone was 18.4 months, compared with 13.9 months for those who received CN followed by sunitinib. No significant differences in PFS were observed. However, in the field of mNCCRCC, these issues had not been addressed. In the current study, we found that both the CN and metastsectomy groups showed significantly better CSS than the non‐CN and non‐metastatectomy groups in Kaplan‐Meier survival analysis (Supplemental Figures 1 and 2); however, no further differences were found between the two groups in terms of first‐line PFS and total PFS. Further randomized clinical trials regarding the role of CN and metastasectomy in the field of mNCCRCC are warranted to provide more definitive results of these issues.

Presently, multivariable Cox regression analysis revealed that liver metastasis was the only significant prognostic factor for both first‐line PFS (HR: 1.856; 95% CI: 1.228‐2.805, *P* = 0.0033; Table 1) and CSS (HR: 3.868; 95% CI: 1.713‐8.205, *P* = 0.0007; Table 2). In the study of IMDC of 2027 patients with metastatic RCC, McKay et al^28^ reported that the presence of bone and liver metastasis in patients treated with TT confers a poor prognosis and aggressive RCC subclones tend to spread to these sites. They also demonstrated that liver metastasis was a poor prognostic factor in both PFS and OS. However, owing to the rarity of liver metastasis, a large cohort or randomized prospective study is not feasible, and the mechanism thus remains elusive. A few previous studies have hypothesized that liver metastasis occurs in association with metastases to other sites, which is in accordance with the hematogenous spread pattern observed in RCC.^29^ In fact, the incidence of “solitary” liver metastases in patients with mRCC has been estimated as only 2%‐4%.^29^, ^30^ Consequently, the burden of hepatic tumors could represent a rate‐limiting step in terms of survival outcomes.

The current study has inevitable limitations. First, even with a large registry study design, our cohort is still relatively small due to the rarity of mNCCRCC. In addition, due to the retrospective nature, the current study is based on a highly heterogeneous study cohort (Supplemental Table S1). Another limitation of the study is the lack of a central review of pathology; subsequently, misclassification of some tumors might have affected survival outcomes. However, to the best of our knowledge, this is the largest study of mNCCRCC in an Asian population, and it can serve as support for previous results in Western populations.^5^, ^6^, ^19^ Also, the current study included the largest number of patients to date for comparing the efficacy of temsirolimus with other agents in mNCCRCC patients as first‐line treatment.^22^ Further larger studies are warranted to validate and generalize our results.

## 5 CONCLUSIONS

Even with significant improvement of survival, for the majority, survival in mNCCRCC patients is still inferior to that in mCCRCC patients in the current TT era. We found survival benefits in patients treated with first‐line VEGF‐TKIs/CN/metastasectomy and MM patients. In addition, we demonstrated several prognostic factors for survival. Further larger studies and randomized clinical trials are needed to verify these findings.

## CONFLICTS OF INTEREST

The authors have no conflicts of interest to disclose.

## Data Availability

The data that support the findings of this study are available from the corresponding author upon reasonable request.
