# Supplementary material for: Survival and clinical prognostic factors in metastatic non‐clear cell renal cell carcinoma treated with targeted therapy: A multi‐institutional, retrospective study using the Korean metastatic renal cell carcinoma registry
Source: Cancer Med. 2019 May 9;8(7):3401–10. doi: 10.1002/cam4.2222 (PMC6601573; doi:10.1002/cam4.2222)

Supplemental Table 1. Baseline characteristics of patients with metastatic non-clear cell renal cell carcinoma

	Number of patients (Total N=156)
Age, yrs, median (IQR)	57 (46-67)
Sex, male, n (%)	108 (69.2%)
KPS, n (%)	
≥90	135 (86.5%)
80	11 (7.1%)
≤70	1 (0.6%)
Unknown	9 (5.8%)
ECOG, n (%)	
0	56 (35.9%)
1	79 (50.6%)
≥2	12 (7.7%)
Unknown	9 (5.8%)
Histology, n (%)	
Papillary	93 (59.6%)
Chromophobe	20 (12.8%)
Collecting duct	18 (11.5%)
Unclassified	16 (10.3%)
Xp11.2 translocation	9 (5.8%)
Type of first-line therapy, n (%)	
Cytokines	14 (9.0%)
VEGF-TKIs	79 (50.6%)
Sunitinib	53 (67.1%)
Sorafenib	10 (12.7%)
Pazopanib	15 (19.0%)
Axitinib	1 (1.2%)
mTORi	63 (40.4%)
Everolimus	16 (25.4%)
Temsirolimus	47 (74.6%)
IMDC risk group at start of 1st-line therapy, n (%)	
Favorable	36 (23.2%)
Intermediate	91 (58.7%)
Poor	28 (18.1%)
MSKCC risk group at start of 1st-line therapy, n (%)	
Favorable	37 (23.9%)
Intermediate	96 (61.9%)
Poor	22 (14.2%)
Type of metastasis, n (%)	
Synchronous	76 (48.7%)
Metachronous	80 (51.3%)
Site of metastasis, n (%)	
Lung	71 (46.1%)
Liver	41 (26.6%)
Lymph nodes	82 (53.3%)
Bone	66 (42.9%)
Brain	5 (3.3%)
Cytoreductive nephrectomy, yes, n (%)	93 (59.6%)
Metastasectomy, yes, n (%)	30 (19.5%)
Surgery	29 (18.6%)
Lung	20 (12.8%)
Liver	4 (2.6%)
Bone	5 (3.2%)

Radiotherapy	15 (9.6%)
Brain (Gamma Knife Surgery)	3 (1.9%)
Bone	12 (7.7%)

Supplemental Table 2. Event and median survival time according to the histologic subtypes

	Clear cell (1768)	Papillary (92)	Chromophobe (19)	Collecting duct (18)	Unclassified (16)	Xp11.2 transposition (9)	Log-rank, P
**First PFS**							
Non-event	517 (34.67)	22 (23.91)	6 (31.58)	7 (38.89)	7 (43.75)	4 (44.44)	
Event	974 (65.33)	70 (76.09)	13 (68.42)	11 (61.11)	9 (56.25)	5 (55.56)	
Median time (95% CI)	8.00 (8.00-9.00)	4.00 (3.00-5.00)	10.00 (4.00-16.00)	4.00 (1.00-16.00)	4.00 (1.00-70.00)	18.00 (0.00-NA)	0.0019
**Total PFS**							
Non-event	492 (32.69)	21 (22.83)	6 (30.00)	7 (38.89)	7 (43.75)	4 (44.44)	
Event	1013 (67.31)	71 (77.17)	14 (70.00)	11 (61.11)	9 (56.25)	5 (55.56)	
Median time (95% CI)	12.00 (11.00-13.00)	6.00 (3.00-8.00)	14.00 (4.00-24.00)	4.00 (1.00-16.00)	4.00 (2.00-82.00)	24.00 (0.00-48.00)	<0.0001
**CSS**							
Non-event	588 (39.12)	27 (29.03)	9 (45.00)	7 (38.89)	3 (18.75)	5 (55.56)	
Event	915 (60.88)	66 (70.97)	11 (55.00)	11 (61.11)	13 (81.25)	4 (44.44)	
Median time (95% CI)	31.00 (29.00-34.00)	19.00 (14.00-27.00)	58.00 (22.00-109.00)	35.00 (6.00-81.00)	10.00 (6.00-14.00)	31.00 (2.00-32.00)	0.0030

Supplemental Figure 1. Kaplan-Meier survival curves for (A) first-line progression-free survival (PFS), (B) total PFS, and (C) cancer-specific survival (CSS) according to the use of cytoreductive nephrectomy

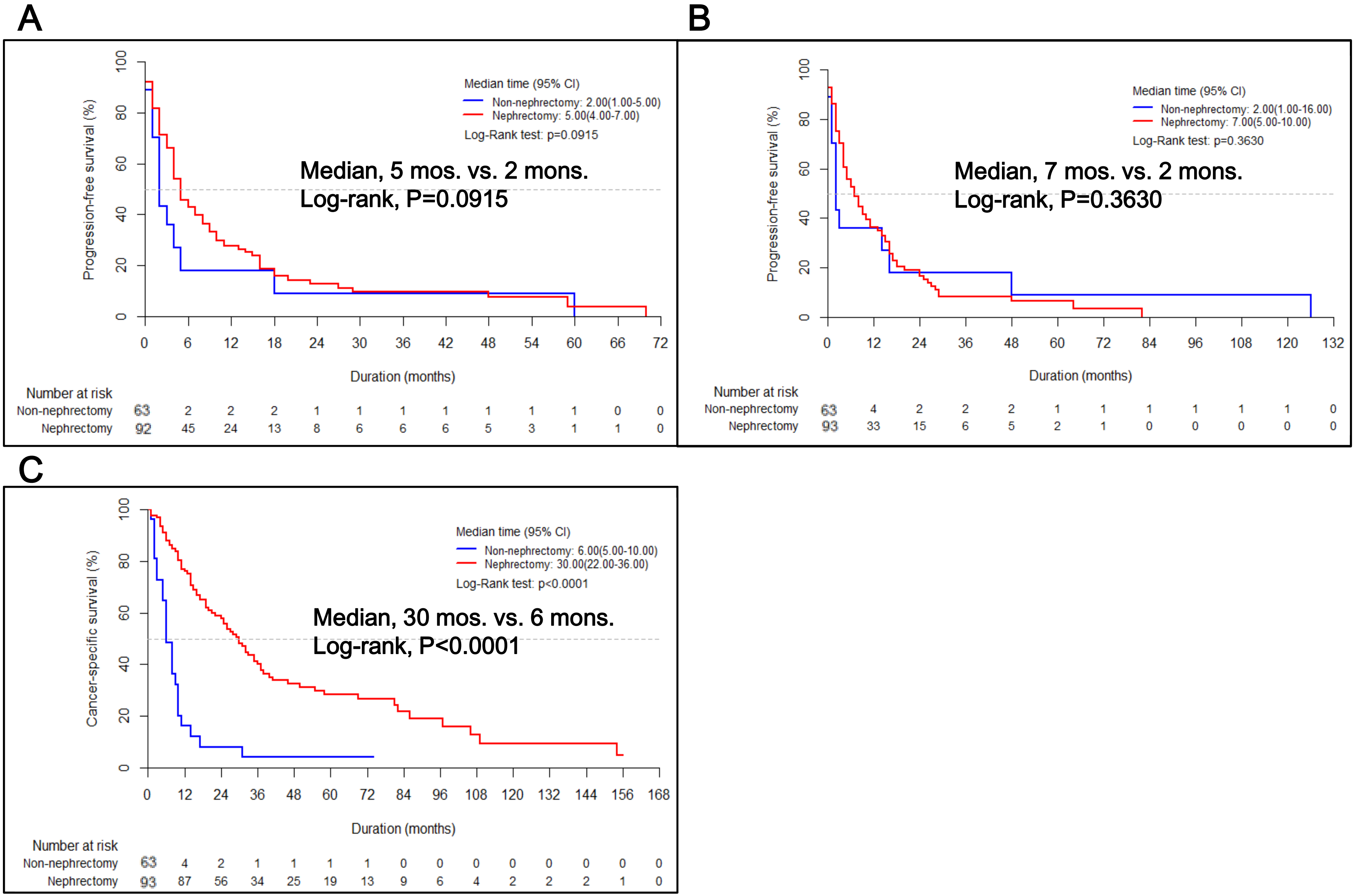

Supplemental Figure 2. Kaplan-Meier survival curves for (A) first-line progression-free survival (PFS), (B) total PFS, and (C) cancer-specific survival (CSS) according to the use of metastasectomy

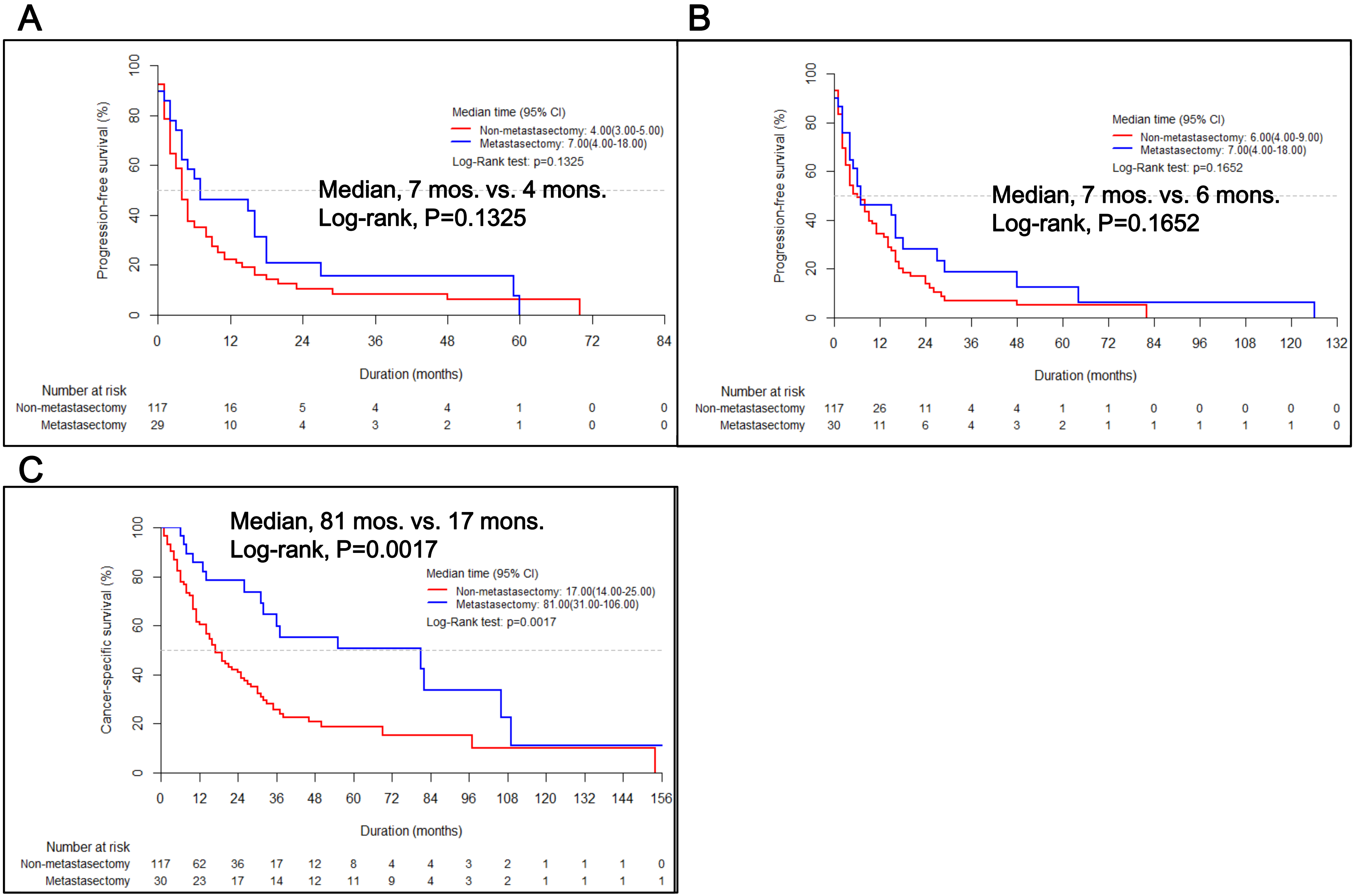

Supplement: Supplementary file 1 [file CAM4-8-3401-s001.pdf]
